# Changes in location and number of nurse consultations as the supply of general practitioners decreases in primary health care: six-year register-based follow-up cohort study in the city of Vantaa, Finland

**DOI:** 10.1080/02813432.2024.2375548

**Published:** 2024-07-08

**Authors:** Aina Enckell, Merja K. Laine, Hanna-Maria Roitto, Marko Raina, Timo Kauppila

**Affiliations:** a Department of General Practice and Primary Health Care, University of Helsinki and Helsinki University Hospital, Helsinki, Finland; b Western Uusimaa Wellbeing Services County, Finland; c Folkhälsan Research Centre, Helsinki, Finland; d Clinics of Internal Medicine and rehabilitation, Department of Geriatrics, University of Helsinki, and Helsinki University Hospital, Helsinki, Finland; e Population Health Unit, Finnish Institute for Health and Welfare, Helsinki, Finland; f Wellbeing Services County of Vantaa and Kerava, Vantaa, Finland; g Apotti Ltd

**Keywords:** emergency department, follow-up, general practitioner, office-hour care, practical nurse, primary health care, registered nurse

## Abstract

**Objective:**

To investigate whether the location and the number of nurse consultations have changed in response to the continuously decreasing number of GP consultations in the fourth-largest city in Finland. It has been suggested that nurse consultations are replacing GP consultations.

**Design:**

A retrospective register-based follow-up cohort study.

**Setting:**

Public primary health care in the City of Vantaa, Finland.

**Subjects:**

All documented face-to-face office-hour consultations with practical and registered nurses, and consultations with practical and registered nurse in the emergency department of Vantaa primary health care between 1 January 2009 and 31 December, 2014.

**Main outcome measures:**

Change in the number of consultations with practical and registered nurses between 2009 and 2014 in primary health care both during office-hours and in the emergency department.

**Results:**

Over the follow-up period, the monthly median number of practical nurse consultations in the emergency department per 1000 inhabitants increased from 1.6 (interquartile range [IQR] 1.3–1.7) to 10.5 (10.3–12.2) (*p* < 0.001) and registered nurse consultations from a median of 3.6 (3.0–4.0) to 14.5 (13.0–16.6) (*p* < 0.001). However, there was no significant change in the median monthly number of office-hour consultations with practical or registered nurses.

**Conclusions:**

It appears that in primary health care, medical consultations have shifted from GPs to nurses with lower education levels, and from care during office-hours to emergency care.

## KEY POINTS

The number of general practitioner (GP) consultations are decreasing. Tasks are being transferred from GPs to nurses to improve access to care.

- The number of office-hour consultations with nurses did not change, despite the decrease in GP consultations.
- In the emergency department, the number of nurse consultations increased significantly when GP consultations decreased.
- Medical consultations seem to have shifted to the emergency department and the nurses.

## Introduction

Adequate access to care is a central part of well-functioning primary health care (PHC) [1,2]. In contrast to the situation in its neighbouring countries [3,4] in Finland general practitioner (GP) consultations have been decreasing in PHC in recent years [3–6]. This trend has been considered desirable to some extent. For instance, in 2008, in response to overload, the City of Vantaa, Finland, initiated a ‘reverse triage’ approach to reduce the utilisation of PHC emergency department (ED) GP consultations [7]. The goal was that patients would be redirected from the PHC ED to office-hour consultations [8]. This reverse triage intervention was successful as the yearly GP consultations in ED per 1000 inhabitants had decreased from 224 to 162 by 2014 [8]. This meant a total decrease of almost 27% [7]. Contrary to expectations, a decrease in the number of office-hour GP consultations was also observed: from 2008 to 2014, GP consultations decreased from 1068 per year per 1000 inhabitants to 885 [9]. Furthermore, the decreasing trend in GP consultations seemed to continue, despite attempts to change it through organisational reforms such as the service provider model change that attempted to ensure sufficient non-urgent office-hour consultations for the most vulnerable populations [10].

It has been suggested that nurses could replace GPs in PHC if there is a shortage of GPs [11,12], to improve access to care [13,14]. PHC nurse consultations could replace GP consultations in both urgent and non-urgent issues [12,15–17] and could also be more cost-effective [18,19]. Therefore, the use of nurses in PHC is expanding [20].

In public PHC in Finland, nurses of various categories work with and alongside GPs: practical nurses, registered nurses and public health nurses. Practical nurses are trained in institutions providing upper vocational education. Their programmes last two to three years and are worth 180 European Credit Transfer and Accumulation System (ECTS) course credits. Registered nurses pursue their education at a University of Applied Sciences, on programmes that last about three and a half years and comprise 210 ECTS course credits. Public health nurses undergo longer programmes of about four years at a University of Applied Sciences and accrue 240 ECTS course credits. In PHC, practical and registered nurses often treat sick patients in PHC settings, whereas public health nurses primarily work in preventive health care, such as in maternity and child health clinics and schools.

The aim of the study was to examine whether the location and the number of monthly patients’ consultations with practical and registered nurses both during public PHC office hours and in ED settings have changed as patients’ GP consultations have decreased.

## Materials and methods

### Setting

This retrospective register-based follow-up cohort study was conducted in the public PHC of the City of Vantaa, Finland. Vantaa is the fourth largest city in Finland with a population of about 220 000 inhabitants. In Finland, health centres that offer the public office-hour PHC are funded and maintained by municipalities, *via* taxes. In Vantaa, the PHC ED in the Peijas hospital was outsourced to a private company during the study period but continues to be funded by the City. People can also use the private sector’s health care services, the costs of which they cover either themselves or *via* voluntary insurance, or the costs are paid by their employer.

In this study, we studied practical and registered nurses’ face-to-face patient consultations in all of Vantaa’s PHC centres and ED, which at the time provided acute PHC services around the clock. Even though ED was outsourced to a private company, their data were recorded in the electronic health record system of the City of Vantaa. The data were gathered from 1 January, 2009, to 31 December, 2014. We chose this period because at the beginning of 2009 every unit in public PHC was computerised and using an electronic health care system. The study was set to end in 2014 because specialised health care was to take over all ED activities in Vantaa. In this study, we focused on practical and registered nurses’ consultations. As public health nurses are responsible for only preventive care in PHC, including maternity and child health clinics and schools, we did not include them in this study. In 2009, there were 94 GPs working in Vantaa’s public PHC, and by 2014, the number had increased to 114. In the outsourced PHC ED between 2009 and 2014, the number of GPs remained unchanged [7].

### Data

Data on the size of the population and the numbers of official practical and registered nurses employed by the City of Vantaa were provided by the Vantaa City administration. The number of practical and registered nurses working in PHC ED services was not available. Data on the consultations were obtained from Vantaa’s electronic health record system (Graphic Finstar-patient chart system, GFS, Logica Ltd, Helsinki, Finland). We collected data on the number of practical and registered nurses’ monthly face-to-face patient consultations and on whether they took place during office-hours or at the ED, which provided care around the clock.

### Outcome

Our primary outcome measure was the monthly number of practical and registered nurse consultations during PHC office-hours and at the ED between 2009 and 2014.

### Ethical aspects

The register keepers, the health authorities of Vantaa and the scientific ethical board of Vantaa (TUTKE), granted permission (VD/8059/13.00.00/2016) to carry out the study. This study was conducted directly using the patient register and no individual patients or nurses were identified. According to the Finnish law on register studies (https://rekisteritutkimus.wordpress.com/luvat-ja-tietosuoja/), our study participants were not required to sign a Statement of Informed Consent because the study was retrospective, based on patient charts, and the investigators did not contact the patients.

### Statistical analysis

The number of nurse consultations were analysed using the Kruskal–Wallis test (nonparametric ANOVA) and repeated measurements (RM-ANOVA), followed by Tukey’s post-hoc test. These tests were chosen due to the nature of data; as the number of consultations varied across different months. Consequently, comparisons between years had to be conducted by comparing corresponding months. The results were expressed as medians and interquartile ranges (IQR) from 25% to 75%. The rate of change, that is, the trends in the numbers of practical and registered nurse consultations, was analysed using regression analysis (GLM procedure of SigmaPlot 10.0 Statistical Software, Systat Software Inc, Richmond, CA, USA). The GLM model enabled us to calculate the mean slope (cofactor a) of the development of the monthly number of the office-hour and ED patient consultations with nurses per 1000 inhabitants, along with its standard error of the mean (SEM). Then comparisons were performed using the t-test to detect whether the slope increased or decreased statistically significantly. This allowed trend analysis to determine the direction in which the monthly consultation numbers were developing.

## Results

At the beginning of the follow-up in 2009, Vantaa had a population of 195,397 inhabitants, with 550 practical nurses and 293 registered nurses working in the PHC centres. By the end of the follow-up in 2014, the population had increased to 208 098, and the number of practical nurses and registered nurses had risen to 594 and 339, respectively (Table 1). Table 1 shows the number of inhabitants and practical and registered nurses who were working in PHC centres in the City of Vantaa during the follow-up period.

**Table 1.** Number of inhabitants and practical and registered nurses working in the primary health care centres of the City of Vantaa, Finland between 2009 and 2014.

Year	Inhabitants (*n*)	Practical nurses (*n*)	Registered nurses (*n*)
2009	195 397	550	293
2010	197 636	580	315
2011	200 055	597	314
2012	203 001	609	306
2013	205 312	588	328
2014	208 098	594	339

The number of practical and registered nurse consultations per month varied significantly between months (*p* < 0.001). In comparison to the busiest month, November, in which the median was 111.5 (IQR 96.2–124.9) consultations per 1000 inhabitants, the numbers of all nurse consultations were lower during the summer months: in June the median was 68.9 (IQR 59.7–72.2) consultations per 1000 inhabitants (*p* < 0.001 vs November), and in July the median was 62.0 (IQR 55.4–65.4) consultations per 1000 inhabitants (*p* < 0.001 vs November).

During the follow-up period, the number of monthly practical nurse office-hour consultations decreased from a median of 3129 (IQR 2689–3468) in 2009 to a median of 2628 (IQR 2343–4117) in 2014 (for trend *p* = 0.058), while ED consultations increased from a median of 311 (IQR 248–341) to a median of 2192 (IQR 2136–2536) (for trend *p* < 0.001). The number of registered nurses’ office-hour consultations decreased from a median of 9150 (IQR 7990–9980) to a median of 8923 (IQR 7720–9110) (for trend *p* = 0.331) whereas the number of ED consultations increased from a median of 703 (IQR 586–782) to a median of 3107 (IQR 2711–3462) (for trend *p* < 0.001). Table 2 presents the median (IQR) of monthly office hours and ED consultations with practical and registered nurses per 1000 inhabitants during the follow-up period. The monthly number of ED consultations of practical and registered nurses increased from 2011 (in comparison to 2009, *p* < 0.001).

**Table 2.** Median number of monthly office-hour and emergency department consultations with practical and registered nurses per 1000 inhabitants in the City of Vantaa, Finland between 2009 and 2014. The statistical comparisons used the corresponding figures from 2009.

	Practical nurses						Registered nurses					
Year	All consultations	*p*-value*	Office-hour consultations	*p*-value*	Emergency department consultations	*p*-value*	All consultations	*p*-value*	Office-hour consultations	*p*-value*	Emergency department consultations	*p*-value*
2009	17.7 (15.5–17.1)		16.0 (13.8–17.8)		1.6 (1.3–1.7)		50.5 (44.7–54.7)		46.8 (40.9–51.1)		3.6 (3.0–4.0)	
2010	17.0 (15.9– 18.6)	ns	15.7 (14.3–16.8)	ns	1.6 (1.4–1.8)	ns	53.8 (47.9–60.5)	ns	50.3 (44.5–57.1)	ns	3.0 (2.7–3.1)	ns
2011	23.7 (20.6–25.6)	<0.001	12.3 (11–13.3)	0.006	11.2 (9.9–12.5)	<0.001	57.0 (52.6–64.7)	0.006	46.8 (44.5–54.6)	ns	9.8 (8.2–10.7)	<0.001
2012	21.7 (19.9–23.9)	<0.001	8.7 (7.9–10.6)	<0.001	12.9 (11.8–14.0)	<0.001	58.9 (52.1–60.6)	ns	45.0 (40.9–48.0)	ns	11.8 (10.9–13.7)	<0.001
2013	25.5 (24.5–27.2)	<0.001	12.1 (10.9–13.0)	0.006	13.1 (12.8–14.4)	<0.001	54.1 (48.8–62.7)	ns	42.9 (35.2–49.7)	ns	12.2 (10.8–13.4)	<0.001
2014	25.2 (22.7–30.8)	<0.001	12.6 (11.3–19.8)	ns	10.5 (10.3–12.2)	<0.001	56.5 (52.1–59.9)	0.038	42.9 (37.1–43.8)	ns	14.5 (13.0–16.6)	<0.001

*compared to 2009, Tukey test. ‘ns’: not statistically significant, medians (interquartile range 25%–75%) are presented.

**Figure 1.**
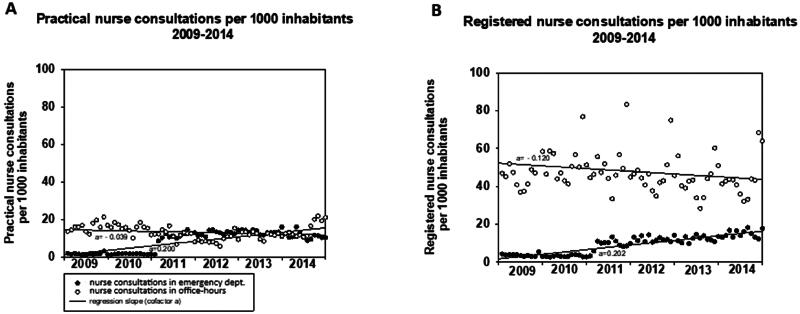
Presents the raw values and regression slopes of monthly office-hour consultations and consultations with registered (A) and practical (B) nurses in emergency department.

Figure 1 presents the rates of change in office-hour consultations and ED consultations with practical and registered nurses. No significant changes were observed in the rate of change in office-hour consultations with practical or registered nurses. The rate of change in monthly ED consultations (cofactor a) was 0.200 per 1000 inhabitants (SEM 0.018) for practical nurse consultations (*p* < 0.001) and 0.202 (SEM 0.012) for registered nurse consultations (*p* < 0.001).

## Discussion

During the six-year follow-up period, no significant change was observed in the number of office-hour consultations with practical or registered nurses in public PHC. However, the number of PHC ED nurse consultations increased significantly: practical nurse consultations increased from a median of 1.6 to 10.5 per 1000 inhabitants and registered nurse consultations increased from a median of 3.6 to 14.5 per 1000 inhabitants. In practical terms, this means an additional 42 consultations with practical nurses per month and 41 consultations with registered nurses per month in the PHC ED setting. Over the same time frame, public PHC GP office-hour consultations decreased by almost one-fourth and ED consultations decreased by almost one-fifth [9].

We observed a yearly recurring monthly variation in the number of nurse consultations: summer months were systemically less busy than winter months, with the number of consultations being the lowest in July-August and the highest in November. This may be due to summer vacations and the fact that in Finland, there are no national holidays or school holidays in November.

In 2011, there seemed to be an abrupt increase in nurse consultations in the PHC ED. We can only speculate whether this was related to the transition in 2011 from ‘the named GP model’ to the restricted-list GP model’ [10], and that the new service model, along with the lack of a regular GP for many patients, may have forced patients to seek help at the PHC ED. We did not observe any change in nurse consultations during office hours. We lack data on whether the nurse workforce in the PHC ED increased at this time or if the same number of nurses had to handle more patients. There was no change in the number of GPs working in the PHC ED [7].

This study had several strengths. The data were comprehensive, encompassing documentation of all face-to-face consultations between both practical and registered nurses and patients in the public PHC of the City of Vantaa, Finland. The number of consultations was substantial, and the follow-up period was long enough to ensure reliable results regarding the observed changes. The recorded consultations accurately represented the actual consultations in terms of both number and location, and the data are considered reliable. Both municipality-employed nurses and company-employed nurses recorded the consultations in the same way, using the same electronic health record system. However, we cannot guarantee that all the consultations were systematically registered and in accordance with the given instructions. Although data were over 10 years old, replacing GP’s work with nurses in PHC is still a topic that deserves attention and evokes discussion [11–20].

Some other limitations must also be taken into account. First, the ED provider was unable to provide data on the exact numbers of practical and registered nurses working in the outsourced PCH ED services during the study period. Consequently, we were unable to track the changes in the numbers of monthly consultations per nurse, which means we were also unable to assess the potential changes in the workload of individual nurses. As we lacked data on private sector consultations, we do not know whether patient treatment moved there. Another limitation was that no information on possible changes in patient characteristics or alterations in the management of consultations and diseases was available. We also had no information on the quality of the consultations or whether the treatment provided by the different types of nurses varied. However, it is known that mortality rates in Vantaa did not change during the study period [7,21], and previous studies have indicated rare occurrences of patient errors during consultations with nurses [22,23]. Due to the retrospective nature of our study, data entered into the database were not collected for research purposes. Since data were not collected in a predesigned format, some data might be missing. Also, certain variables could potentially impact the outcome may not have been recorded. Finally, when considering the significance of the results, it should be kept in mind that the study data were collected from 2009 to 2014.

Most studies of nurses replacing GPs have reported that physicians are replaced by and compared with highly educated nurse practitioners [18,24]. The term nurse practitioner is generally used for registered nurses who have completed further education, such as a master’s degree in advanced nursing practice. Nurse practitioners operate within an extended scope of practice, and are qualified to give diagnoses, prescriptions, and treatment for medical conditions [25]. A previous study has reported that nurse practitioner services positively impact the quality of care, patient satisfaction, and waiting times in the ED [26]. However, this study suggests a different trend in the City of Vantaa. From an administrative perspective, the replacement of the workforce with less educated health care professionals could potentially lead to cost savings [18,19].

The findings of our study raise concerns, as the decrease in GP consultations does not seem to have shifted the workload to nurses’ office-hour consultations. On the contrary, nurse consultations in the ED appear to be increasing. Our study findings show that public PHC office-hour consultations are shifting to ED, and to less educated health care professionals. Furthermore, the present data suggest that there is no automatic transfer of GP tasks to nurses in PHC office-hours services if the number of GP appointments decreases. If PHC organizations plan to shift GP tasks to nurses, careful planning is necessary; otherwise, patients may end up in EDs, which are already prone to overcrowding.

The shift of the focus of care to ED poses several challenges, such as higher costs and poorer continuity of care than that of office-hour consultations [27]. The reverse triage procedure itself would not lead to a constant increase in ED nurse consultations if office-hour GP services were adequately maintained [28]. Previous observations have also indicated that the reduced number of GPs is associated with a reduction in the time spent with patients [12]. To ensure continuity of care, which is essential due to its many benefits, also in nurse-led care [29] office-hour care seems to be more beneficial to patients. This is even more important now when there will be further reductions in resources of the Finnish PHC due to government policy. In the current situation, with limited GP and nurse resources and an aging population, it is crucial that patients are treated in a safe, appropriate, and cost-effective manner.

Further studies are needed to determine whether shifting tasks to nurses is a safe and cost-beneficial option in the holistic care of patients in PHC settings. Further studies are needed to provide more insights into recent shifts in nurse consultations. Additionally, further studies are needed to evaluate the factors and reasons behind the decrease in the number of GP consultations in public PHCs.
